# Problem-solving approach to continuing health education in nursing training: an experience in hospital care

**DOI:** 10.1590/1980-220X-REEUSP-2021-0543en

**Published:** 2022-06-24

**Authors:** Valentina Barbosa da Silva, Aldrin de Sousa Pinheiro, Lerissa Nauana Ferreira, Izabela Viegas Cunha, Rayssa Thamyres Menezes Cavalheiro, Marluci Andrade Conceição Stipp

**Affiliations:** 1 Universidade Federal de Rondônia, Departamento de Enfermagem, Porto Velho, RO, Brazil.; 2 Universidade Federal do Rio de Janeiro, Escola de Enfermagem Anna Nery, Rio de Janeiro, RJ, Brazil.

**Keywords:** Education, Continuing, Health Policy, Health Strategies, Education, Professional, Hospitals, Educación Continua, Política de Salud, Estrategias de Salud, Educación Profesional, Hospitales, Educação Continuada, Política de Saúde, Estratégias de Saúde, Educação Profissionalizante, Hospitais

## Abstract

**Objective:**

To report the experience of an educational practice of sensitization of health workers to the actions of Permanent Health Education.

**Method:**

This is an experience report, of a qualitative and descriptive nature, conducted through the Arc of Maguerez, in 2019, in a reference hospital for the treatment of infectious diseases in the state of Rondônia.

**Results:**

The Arc of Maguerez was an important point for intervention in the context reality, through two dynamics entitled “myths and truths” and “ludic wheel”, which facilitated reflection and understanding of the role of continuing education in the services studied.

**Conclusion:**

Continuing education actions play a fundamental role in the construction of expertise, expanding knowledge, as they facilitate the sharing of new experiences with the team and the external public.

## RESUMO

**Objetivo:**

Relatar a experiência de uma prática educativa de sensibilização dos trabalhadores de saúde às ações de Educação Permanente em Saúde.

**Método:**

Trata-se de um relato de experiência, de cunho qualitativo e natureza descritiva, conduzido por meio do Arco de Maguerez, no ano de 2019, em um hospital de referência para o tratamento de doenças infectocontagiosas do estado de Rondônia.

**Resultados:**

O Arco de Maguerez foi ponto importante para a intervenção na realidade do contexto, por meio de duas dinâmicas intituladas “mitos e verdades” e “roleta lúdica”, que facilitaram a reflexão e a compreensão sobre o papel da educação permanente nos serviços estudados.

**Conclusão:**

As ações de educação permanente têm papel fundamental na construção de saberes, ampliando o conhecimento, por facilitar o compartilhamento de novas experiências com a equipe e o público externo.

## RESUMEN

**Objetivo:**

Relatar la experiencia de una práctica educativa de sensibilización de los trabajadores de salud a las acciones de Educación Permanente en Salud.

**Método:**

Se trata de un relato de experiencia, del tipo cualitativo y naturaleza descriptiva, conducido por medio del Arco de Maguerez, en 2019, en un hospital de referencia para tratamiento de enfermedades infectocontagiosas del estado de Rondonia, Brasil.

**Resultados:**

El Arco de Maguerez fue punto importante para la intervención en la realidad del contexto, por medio de dos dinámicas llamadas “mitos y verdades” y “ruleta lúdica”, que facilitaron la reflexión y la comprensión sobre el rol de la educación permanente en los servicios estudiados.

**Conclusión:**

Las acciones de educación permanente tienen un rol fundamental en la construcción de saberes, ampliando el conocimiento, pues facilitan el compartir de nuevas experiencias con el equipo y el público externo.

## INTRODUCTION

In the hospitals field of action, often marked by the influence of the hegemonic model tradition, health workers face challenges when working with models favoring significant changes in their practices^(1)^.

Permanent Health Education (*EPS*) is a political-pedagogical strategy developed internationally by the Pan American Health Organization (PAHO)^(1)^ that aims to reduce the gaps in the fragmented and, several times, individualized training, for a health worker’s education that contributes to integrated qualifications of the situations contextualized by the professionals themselves. Such an initiative presupposes collective work actions, bringing together the social, political, economic, and historical dimensions of men and women in the labor world^(2)^.

In this article, we adopt Paulo Freire’s definition of Permanent Education (PE), based on a pedagogy allowing autonomy and the construction of men and women in the world. The author Freire considers all dimensions of the individuals, who are capable of teaching and learning in their relationships mediated by dialogue, transforming their lives and destiny in a collective and collaborative way^(2)^.

*EPS* was instituted in Brazil through the National Policy on Permanent Health Education (*PNEPS*), Ministerial Ordinance No. 1996 of August 20, 2007, which also began to indicate the relationships between training and management, institutional development and social control in health^(3,4)^. *EPS* seeks to overcome the traditional models of qualification and continuing education, in which activities were designed and developed without considering the services real needs^(5)^.

*EPS* is an essential instrument for collective construction, using problematization and reflections about the work process and its experiences, in addition to being based on meaningful learning and on the perspective of transforming professional practices. The theoretical bases of *EPS* are autonomy, citizen participation, actors’ subjectivity, and learning in/through/for health work daily practice. It shall take place jointly within health workers, students, and users^(6)^.

In the initial health workers’ training, the inclusion of theoretical-conceptual and methodological elements of *EPS* is essential. In this study, the implementation of educational practices developed in collaboration with health service workers is highlighted. In this regard, an investment was made in the inclusion of students of an undergraduate nursing course in the daily life of health services, so that the experience/experimentation with workers and managers allowed significant learning, considering the reality of the world of work in the Brazilian Public Health Care System (*SUS*).

The educational activity was initiated after the discussion and identification of the problem reported by the components of the Educational Practice Working Group (*GTPE*), which consisted of students and professors from the Universidade Federal do Estado de Rondônia (*UNIR*), as well as a nurse who was the coordinator of the Permanent Education Center (*NEP*) of the Reference Hospital in infectious diseases of the State of Rondônia. *NEP* acts in the work environment of collective construction, in planning with workers for the elaboration of permanent education actions.

Despite *NEP’s* attributions, some obstacles were found for its implementation, such as insufficient human resources in the sectors, which hinders workers from participating in actions during working hours; workers considered resistant to updating; and the lack of articulation among heads of sectors, managers, and staff so as to participate in educational activities^(6)^.

As a central problem, based on the experience reported, the low adherence of workers to the educational actions promoted by the NEP was highlighted. The problem presented was seen as a means to expand learning of both the initial training (undergraduation) and the training of service workers. This way, the aim was to improve pre-existing knowledge and to include professionals, students, and teachers, promoting integration and co-responsibility for patient safety, which directly affects the quality of care provided, valuing the work and the worker’s main role^(7)^. The rationale for the study was the need to implement and share educational strategies anchored in the problematization of health services, adding to the training resulting from undergraduation.

In the search for evidence, little has been discussed about the educational practices experienced in health services daily life that are close to *EPS*’s problematizing elements. Thus, the dissemination of *EPS* strategic and technological innovations carried out during the work process in the various contexts of health care, including the hospital, becomes essential. Given the above, the present study aimed to report the experience of an educational practice to sensitize health workers to *EPS* actions.

## METHOD

### Design of Study

This is an experience report, with a qualitative approach and descriptive nature, about an educational practice anchored to the methodology of problematization^(2,8)^. In the process of collective and problematizing construction, it was important to use the Arc of Maguerez for better understanding of the stages, since students learn something when they transform it: actively participating in the observation of reality. Moreover, the identification of the problem promotes its theorization and the elaboration of practical solutions for the return to reality.

**Figure 1.**
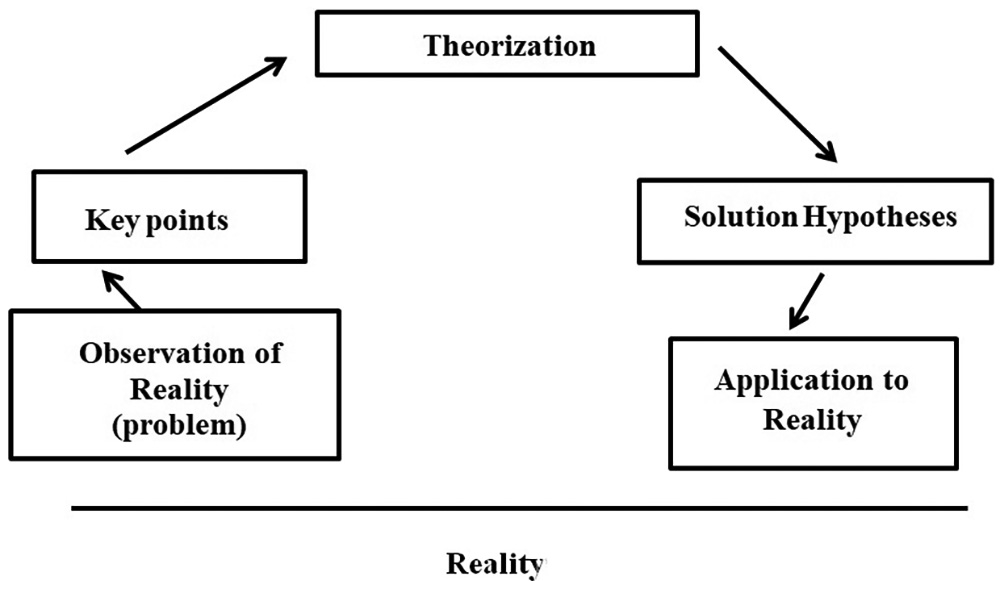
Representative diagram of the Arc of Maguerez.

Source: Bordenave and Pereira (1989)^(8)^.

To illustrate the stages, a diagram (Figure 1) representing an adaptation of the Arc of Maguerez model is presented^(8)^.

In this regard, the following steps were followed: observation of reality, definition of key points, theorization, solution hypotheses, and application to reality in a collaborative way. The experience reported was developed as a stage of the discipline “Educational Practice in Health/Nursing”, from August to October 2019. This is a discipline that comprises EPS theoretical bases in the Undergraduate Nursing Course at UNIR, in the North region of Brazil.

### Local

The reference hospital for the treatment of infectious diseases, in the municipality of Porto Velho, Rondônia, was the place where the experience was developed, but specifically in three internal medicine units, an isolation sector, and an Intensive Care Unit (ICU). This hospital is a High Complexity Care Unit, being used as a practical learning field for UNIR’s health students. From 2020 on, it became a reference for cases of Human Infection by the new Coronavirus (Covid-19).

### Population

The educational practice involved students and professors of the 7th semester of the undergraduate nursing course, who were currently having practical classes on the premises of the Hospital, and health workers (nurses, nursing technicians, and a physiotherapist), who were performing their duties determined by shifts in three internal medicine units, the isolation, and in the ICU.

The study sample was of convenience, consisting of 32 participants: seven nursing students and two professors from *UNIR*, one nurse from *NEP*, and 22 health professionals working in the internal medicine clinics, in the isolation, and in the ICU (16 nursing technicians, 5 nurses, and 1 physiotherapist). It should be highlighted that in the internal medicine clinics, in isolation, and in the ICU, there were 47 potential participants, who were identified through a shift schedule made available by the Administrative Management, and of these, 22 composed the sample to allow the performance of the Intervention Proposal (IP).

The inclusion criteria were: being a student or professor of the 7th semester of *UNIR’s* undergraduate nursing course; health workers, who were in the exercise of their functions determined by shifts in the fifth and sixth meetings organized by the *GTPE*, when the fifth stage of the Arc was developed – the application to reality. Health workers who were on leave or vacation were not included in the study.

### Data Collection

Data collection took place between August and October 2019, in six meetings, considering the stages of the Arc of Maguerez, during round-table discussions, with an average duration of three hours each meeting.

In the first meeting, the initial stage of data collection took place – the observation of reality – triggered by the following question: “What are the main situations the team find in their daily practices?” Based on the participants’ responses, a list of the key points (first and second stage of the Arc of Maguerez) was made. At the end, a question was defined for the theoretical explanation searches. A dispersion period of seven days was established, in which the *GTPE* carried out searches on scientific bases, expanding the understanding of the problem and its key points through theorization.

In the second meeting, the *GTPE* developed the theorization stages and solution hypotheses (third and fourth stages of the Arc of Maguerez) through the return to the situation – main problem, search strategies, and discussion of the findings of the scientific bases, ending with the indication of the construction of an Intervention Proposal (IP). A new dispersion period of seven days was agreed for reflection and the beginning of the construction of the IP, the basis of stage five: the application to reality.

In the third and fourth meetings, the *GTPE* prepared the IP, resulting in a proposal for an educational action to be applied in the sectors previously defined for the activities. The IP was presented and discussed with the nursing and physiotherapy coordinators to broaden the debate, acceptance, feasibility, importance, and need for the development and collaboration of the IP.

In the fifth stage of the Arc – the application to reality –, the use of the constructed IP was the basis, with its implementation being consolidated in the fifth and sixth meetings. At this stage, the *GTPE* was divided into two subgroups, each consisting of four students and a professor and the nurse from the *NEP*. In all meetings, round-table discussions were developed and records registered on the field diary.

At the end of the fifth stage, a moment was reserved for the evaluation of the educational practice, in which the workers were able to express their opinions and perceptions on the following aspects: the methodology used, the time and place of development of the strategy, the relevance of the activity in the context of work, the acceptability about the approach, as well as adherence, awareness, participation and the importance of *EPS* in the hospital environment.

### Data Analysis and Treatment

The results were based on the action-reflection-action process about the reality of the activities developed through the Arc of Maguerez. The contents were recorded in field diaries individually and summarized in a single record. Content organization was carried out right after the meetings and the transcript read as “memories” at the beginning of the subsequent meeting. The study was conducted in accordance with the Consolidated Criteria for Qualitative Research Reports – COREQ^(9)^.

### Ethical Aspects

There was no appreciation by the Research Ethics Committee, based on art. 1 of the Resolution of the National Health Council, No. 510 of 2016^(10)^. It was a report presenting a discussion of the expositions made by the authors of this study, without identifying the participants and ensuring confidentiality and privacy of the experience lived in professional practice.

## RESULTS

The results are presented according to the stages developed, considering the Arc of Maguerez.

In the first stage, Observation of Reality – the *GTPE* was constituted, with seven nursing students, two professors and one nurse (*NEP’s* coordinator). Among the products achieved at this stage, we can highlight the workers’ closer relationship, the ease of dialogue with those working directly with the patient, the management and coordination of actions among the participants, in addition to the expansion of dialogue and care assured, negotiating important situations for acquaintanceship, and reaffirming respect for individual opinions and understandings regarding the unity of the group.

In the second stage, the main key points related to care were listed and grouped (medication errors, poor hand hygiene, difficulties in patient’s extra-hospital transportation, mental health demand, and deficit in the reporting to the *NEP* of the problems identified in the clinics by care workers) and those related to educational actions (team resistance to activities; unavailability of schedules, lack of encouragement from workers to get qualifications, low adherence of workers to existing physical resources, such as the auditorium, and high turnover of duty teams).

**Figure 2.**
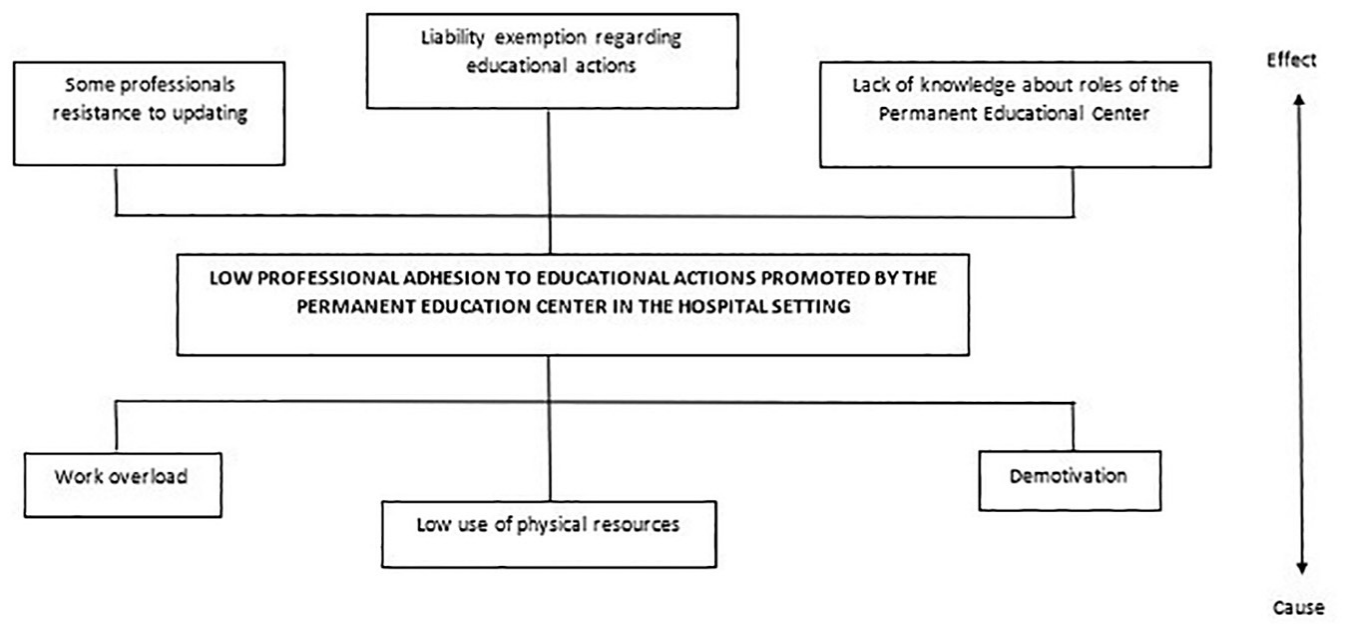
Problem tree.

Source: Prepared by the authors, 2019.

The elaboration of the topics referring to the observation of reality and the finding of key points supported the definition of the central problem, which was the basis for the development of the other stages of the arc. Thus, the workers’ low adherence to the educational actions promoted by *NEP* was considered a priority problematic situation for the development of this experience (Figure 2).

In the third stage, called “theorization”, a search was carried out in the Virtual Health Library (VHL) with the descriptors: Continuing Education, Health Policies, Strategies, Professional Practice and Nursing Education. The selected articles were discussed, understood and summarized, based on the elements, on the different concepts of *EPS*, continuing education and health education, as well as on the comprehension of the performance of *NEP*, relating them to possible reasons for resistance to educational actions in the various contexts of care, from Primary Health Care to hospital care.

In the fourth stage, entitled “solution hypotheses”, four possible elucidations of the central problem were elaborated, and then an intervention proposal was constructed to be applied in reality (step five) based on feasible actions in the setting. The hypotheses that were listed and organized numerically are described below (1–4).

1. The presence of care workers, articulated with *NEP* and with the Centers for Patient Safety, Blood Transfusion, Dressing and other instituted centers, facilitates problem identification and, consequently, enhances intervention actions.
2. Understanding the concepts of *EPS*, health education, continuing education and in-service education promotes workers’ adherence to educational actions in the hospital environment.
3. The difficulties of workers adhering to educational actions are reduced with the expansion of human resources.
4. The following strategies can work as potent alternatives to integrate and sensitize workers to educational actions: *Cine Pipoca*; the reading points; round-table discussions; listening; the dialogue; the flexibility of meetings; education in everyday life; functional progression; *NEP*’s management meetings; the focus group; the protocols; experiences and sharing; the observation; bibliographic research; and the elaboration of standard operating procedures (SOP).

In discussion with the *GTPE*, a consensus was reached that the solution of hypothesis number 4, referring to the fourth stage, would be the most feasible and viable for solving the problem in question. Therefore, this was the solution hypothesis explored in the last stage of the Arc of Manguerez, called “application to reality”. To this end, an IP was constructed, considering the strategies found in the literature^(1–9)^, that stimulate listening, dialogue, flexibility in meetings, experiences and sharing.

Thus, in stage five, the intervention was developed in two moments. Initially through a dynamic entitled “myths and truths”, referring to the function of *NEP* and the understanding of *EPS* and continuing education concepts, using dichotomous responses, such as being myth or truth, in the face of the following sentences:

1. *NEP* is the hospital’s ombudsman, all problems can be reported in this sector to be communicated to the hospital’s management. Result: most workers answered no.
2. *NEP* is responsible for the education of employees, users, and external public. Result: the workers understood that yes, that is, *NEP* can carry out these actions.
3. Continuing education must occur from the problematization of the work process and shall be guided by the user’s health needs. Result: most workers indicated the role of *NEP* as positive and a potentiator of *EPS’s* actions.

**Figure 3.**
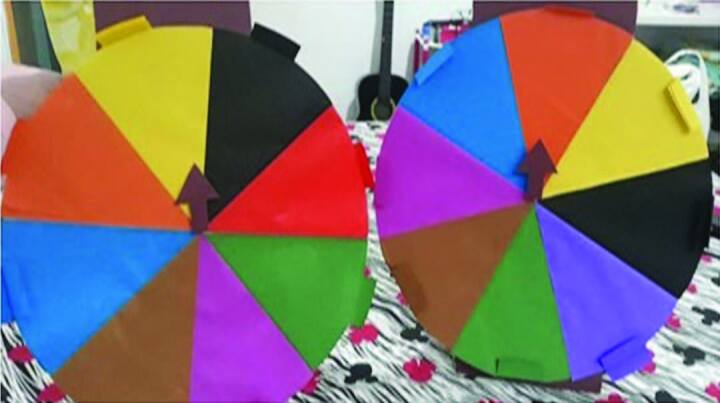
The ludic wheel.

Source: Prepared by the authors, 2019.

In the second moment, the dynamics entitled “ludic wheel” was used, in which the researchers built a ludic and colorful wheel (Figure 3) divided into eight compartments with internal spaces for the description of the different activities in labels that were made manually. The labels were designed and distributed in these eight compartments, allowing each participant to draw a topic through the spin of the wheel. Each label contained educational activities related to the themes discussed by *NEP* and other topics that could also be worked on, even if not yet mentioned by the group, such as: auriculotherapy; cardiopulmonary resuscitation; patient safety; Regulatory Standard 32; talks about safety and health at work in health services; mental health; workers’ health and healthcare-associated infections.

The topic of mental health was highlighted and related to the emotional instability presented by some workers. As one of the strategies to mitigate this situation, the action performed by a hospital nurse, who promoted crochet workshops and auriculotherapy sessions, was highlighted.

Regarding the theme of Patient Safety, structural and work process issues were raised, such as the inadequacy of the bed rails to maintain patient safety, the inappropriate removal of identification bracelets by users, and the discourtesy of patient’s companions and of users with the professionals. They also highlighted the importance of workers’ health, emphasizing the delicate health situation of professionals who deal with the health-disease process of others and of themselves, having to reconcile long and strenuous working hours.

At the evaluation moment, the professionals expressed a positive reaction, praising the relevance of the approach, with emphasis on the performance of participatory educational actions with *NEP’s* coordinating nurse. They stated that they enjoyed the activity conduction, especially during working hours, as it allowed for greater participation. They learned and answered questions during the dynamics, which allowed for greater learning, since such teachings are in line with their daily practices in the hospital environment.

**Figure 4.**
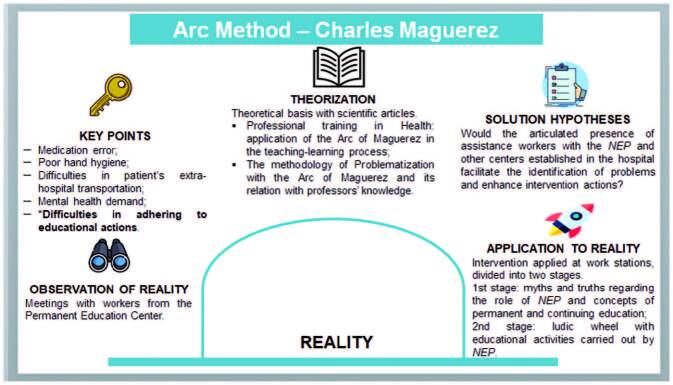
Synthesis of the educational practice construction developed in the hospital environment, according to the stages of the Arc of Maguerez. *Among the points listed by the *GTPE*, this was the priority for the development of the subsequent stages of the Arc of Maguerez.

Source: Prepared by the authors, 2019.

All steps covered – observation of reality, key points, theorization, solution hypotheses and application to reality^(11)^ – are summarized in Figure 4.

## DISCUSSION

The discussion in this report seeks, in fact, a dialogue with the scientific evidence found in the searches, much more concentrated in stage two, regarding theorization, which concerns the possible paths for an educational practice developed in the daily work. In view of the situations resulting from the implementation of the Arc of Maguerez’ stages and the strategies that permeate and facilitate dialogue, investment was made in the formation and conduction of a working group, in dialogic meetings in the sector itself, in the use of dynamics to clarify the most common doubts, as well as in the application of playful instruments, such as the ludic wheel.

Thus, it can be said that the educational practices developed in the work sector improved the group’s ability to critically recognize reality, going beyond the replication of methods, techniques, and knowledge. It means offering individuals the possibility of analyzing their reality in a political and social way, to define joint actions to solve problems and transform health-related situations^(4,11)^. The educational practices that aggregate knowledge and needs of the subjects – the health workers – contribute to their meaningful learning^(2)^.

*GTPE* proved to be an educational strategy from the construction process to the practice with professionals. The group allowed approximation and organization of the work, as well as the development of all stages of the Arc of Maguerez, based on the discussion and exchange of knowledge among the components. This finding is in line with other experiences that used problematization as a path capable of, at the same time, facilitating participation, speaking, listening, organization and also proposing and adequately intervening in reality^(12)^.

The proposed problematization draws attention to decision making based on the investigation of reality, data analysis, definition of priorities, objectives and goals, with the objective construction of actions and evaluation process. This was facilitated by its structuring, composed of the stages presented, articulated to the reality experienced with the theory and the feasible interventions in the work context^(2,8)^.

This way, the managers and health work teams’ governability capacity was expanded to face the problems detected. At all stages, subjects were involved in the teaching and learning process and articulation between theory and practice, which resulted in a renewed, critical, constructivist and liberating praxis^(2,12)^.

Based on statements about myths and truths, the workers reflected on the role of *NEP* in the hospital, as well as discussed the term *EPS* and expressed their doubts regarding the concepts, the attributions of *NEP* and the possibility of considering the Center an ombudsman. They also reflected on the relevance of *EPS* and its interrelationship with professionals, patients, and companions. Most workers emphasized the role of *NEP* in line with the literature^(1,13,14)^, as the sector responsible for providing actions to exchange experiences, through workshops, courses, training, among other strategies.

During the debate on the themes drawn in the dynamics (moment two) of the ludic wheel, an aspect to be highlighted was the positive reaction to the educational actions of the nurse coordinating *NEP*. The educator role was evident as an essential attribution of nurses, as their performance at the Center was transformative by associating education, problematization of the work process, critical reflection and significant learning^(2,11)^. In addition, in the evaluation moment carried out at the end of the dynamics, it was possible to understand the importance of the main themes related to the work context, so that the educational practice attributed meaning and allowed professionals to learn about *EPS* and the educational actions proposed by NEP^(1)^.

At the end of the meetings, it was noticed that the workers responded positively to the methodology and time used, presenting an expanded understanding of the *EPS* from the problematization of the work process and based on the user’s health needs. This understanding is essential in the process of changing practices and indispensable in the sharing of knowledge^(1)^.

There was also good adhesion of the participants and great acceptability of the educational practices applied in collaboration with the authors of this study. This evaluation may be related to the strategies of approximation and insertion in the work reality, facilitating participation in activities^(5)^. The application of dynamics as a teaching and learning strategy allowed the team to discuss and reflect on the importance of *EPS*, considering the educational actions already carried out by *NEP* and allowing the sharing of knowledge^(11)^.

During the educational proposal implementation, the group experienced the feeling of collaboration with the service practice. It was possible to share theoretical knowledge and, above all, to visualize how important the work of NEP is^(1)^. It should be noted that such importance implies constant qualification so that professionals working in this sector have competence and commitment both to work and to workers and users, the focus of care in the hospital unit. This ensures safety for hospital employees, users, companions, and the civil community^(1,5)^.

In addition to demonstrating the need for health workers to welcome *EPS* and support it at the institutions^(11)^, this experience allowed observing the acceptability and participation of workers in the educational actions developed at *NEP*. In fact, when there is pedagogical work with methodological rigor and lightness of the constructive proposal, with possible strategies to add understanding to the participants, the work is much more valued, welcoming and resolute^(2)^. This experience proved to be successful in the hospital environment and an important strategy for the application of a relational technology in EPS, in collaboration with professors, students and health system workers, and should therefore be shared.

## CONCLUSION

*EPS’s* actions prove to be strategies to expand knowledge through the sharing of new technologies and experiences with the team and the external public. The application to the professionals’ reality, made possible by the problematization anchored in the Arc of Maguerez, was relevant thanks to the insertion in the professionals’ routine, and the understanding of the work dynamics in the sectors, in the search to contemplate the largest number of workers. Moreover, it was a good opportunity to contribute to the students’ pedagogical training, as well as to define strategies to enhance the continuing education processes.

The dynamics used during the application to reality, entitled “myths and truths” and “ludic wheel”, proved to be important facilitating technologies with regard to the health workers’ participation and reflection on *EPS* actions. Thus, the dissemination of this report contributes to health workers, especially nursing, since such technologies can promote the workers’ awareness about teaching and learning at work in the hospital area, which can promote the adhesion of workers to the actions developed by *NEP*, demonstrating their opinions, sharing knowledge, re-signifying the attributions of this center on the training processes within the hospital environment.

The non-contemplation of a greater number of workers in the application of the fifth stage of the Arc – application to reality – was a limiting factor, and signals that in new similar initiatives, the moments of intervention occur in extended periods. The educational practice results carried out showed the significance of the teaching-service integration and of the use of problematizing and participatory pedagogical strategies to strengthen the permanent education processes.

## ASSOCIATE EDITOR

Thelma Leite de Araújo
